# Dehydroepiandrosterone (DHEA) supplementation improves in vitro fertilization outcomes of poor ovarian responders, especially in women with low serum concentration of DHEA-S: a retrospective cohort study

**DOI:** 10.1186/s12958-018-0409-z

**Published:** 2018-09-17

**Authors:** Chyi-Uei Chern, Kuan-Hao Tsui, Salvatore Giovanni Vitale, San-Nung Chen, Peng-Hui Wang, Antonio Cianci, Hsiao-Wen Tsai, Zhi-Hong Wen, Li-Te Lin

**Affiliations:** 1 Department of Obstetrics and Gynecology, Kaohsiung Veterans General Hospital, No.386, Dazhong 1st Rd., Zuoying Dist, Kaohsiung City, 81362 Taiwan; 2 Department of Obstetrics and Gynecology, National Yang-Ming University School of Medicine, No. 155, Sec. 2, Li-Nong Street, Pei-Tou, Taipei, 112 Taiwan; 3 Department of Pharmacy and Master Program, College of Pharmacy and Health Care, Tajen University, No.20, Weixin Rd, Yanpu, Township, Pingtung County 90741 Taiwan; 4 Department of General Surgery and Medical Surgical Specialties, University of Catania, 95123 Catania, Italy; 5 Department of Obstetrics and Gynecology, Taipei Veterans General Hospital, No. 201, Section 2, Shih-Pai Road, Taipei, 112 Taiwan; 6 Department of Medical Research, China Medical University Hospital, No. 2, Yude Road, North District, Taichung City, 40447 Taiwan; 7 Department of Marine Biotechnology and Resources, National Sun Yat-sen University, 70 Lienhai Rd, Kaohsiung City, 80424 Taiwan; 8 Department of Biological Science, National Sun Yat-Sen University, 70 Lienhai Rd, Kaohsiung City, 80424 Taiwan

**Keywords:** Dehydroepiandrosterone, DHEA, Diminished ovarian reserve, In vitro fertilization, Poor ovarian responders

## Abstract

**Background:**

Dehydroepiandrosterone (DHEA) is now widely used as an adjuvant for in vitro fertilization (IVF) cycles in poor ovarian responders (PORs). Several studies showed that DHEA supplementation could improve IVF outcomes of PORs. However, most of the PORs do not respond to DHEA clinically. Therefore, the aim of this study is to confirm the beneficial effects of DHEA on IVF outcomes of PORs and to investigate which subgroups of PORs can best benefit from DHEA supplementation.

**Methods:**

This retrospective cohort study was performed between January 2015 and December 2017. A total of 151 PORs who fulfilled the Bologna criteria and underwent IVF cycles with the gonadotropin-releasing hormone antagonist protocol were identified. The study group (*n* = 67) received 90 mg of DHEA daily for an average of 3 months before the IVF cycles. The control group (*n* = 84) underwent the IVF cycles without DHEA pretreatment. The basic and cycle characteristics and IVF outcomes between the two groups were compared using independent t-tests, Chi-Square tests and binary logistic regression.

**Results:**

The study and control groups did not show significant differences in terms of basic characteristics. The study group demonstrated a significantly greater number of retrieved oocytes, metaphase II oocytes, fertilized oocytes, day 3 embryos and top-quality embryos at day 3 and a higher clinical pregnancy rate, ongoing pregnancy rate and live birth rate than those measures in the control group. The multivariate analysis revealed that DHEA supplementation was positively associated with clinical pregnancy rate (OR = 4.93, 95% CI 1.68–14.43, *p* = 0.004). Additionally, in the study group, the multivariate analysis showed that serum dehydroepiandrosterone-sulfate (DHEA-S) levels < 180 μg/dl were significantly associated with a rate of retrieved oocytes > 3 (OR = 5.92, 95% CI 1.48–23.26, *p* = 0.012).

**Conclusions:**

DHEA supplementation improves IVF outcomes of PORs. In PORs with DHEA pretreatment, women with lower DHEA-S level may have greater possibility of attaining more than 3 oocytes.

## Background

Poor ovarian responders (PORs) are the women who respond suboptimally to ovarian stimulation with gonadotropins. While a variety of definitions exist for POR [1], the ESHRE consensus group standardized the definition of POR and established the Bologna criteria in 2011 [2]. It is a great challenge for PORs to reach live birth in in vitro fertilization (IVF) cycles [3, 4]. No certain protocol or single intervention is accepted as an effective method to overcome the poor prognosis of PORs [5]. Therefore, multiple strategies, including various IVF protocols [6], the use of adjuvant supplements [7, 8] and accumulation of vitrified oocytes or embryos [9] have been attempted for PORs undergoing IVF cycles. However, the optimal management for PORs remains an unsolved problem.

Regarding adjuvant supplements, dehydroepiandrosterone (DHEA) is currently widely used worldwide and is considered a potential agent to ameliorate the IVF outcomes of PORs. DHEA is an endogenous steroid generated by the adrenal glands and ovarian theca cells, and acts as a precursor for testosterone [10], which was reported to be engaged in early follicular development [11]. DHEA was first used in PORs in 2000 by Casson et al. who demonstrated that DHEA treatment could enhance response to ovarian stimulation [12]. Later, several studies showed the beneficial effects of DHEA on ovarian reserve [13], oocytes and embryo quality [14, 15] and pregnancy outcomes [16, 17] in PORs. Recent meta-analyses also revealed that DHEA supplementation could improve ongoing pregnancy or live birth in PORs undergoing IVF cycles [18, 19]. However, there is a lack of large-scale, well-designed randomized controlled trials to verify the beneficial effects of DHEA in PORs.

In clinical practice, some PORs saw improvement in IVF outcomes after DHEA supplementation; however, others failed to respond to DHEA treatment. It is believed that there are certain subgroups of PORs who could benefit more from DHEA supplementation than others. Therefore, the aim of this study is to confirm the beneficial effects of DHEA on IVF outcomes and to identify the subgroups of PORs that may benefit more from DHEA supplementation.

## Methods

### Study design

This is a retrospective cohort study. Patients were treated at the Reproductive Center of the Kaohsiung Veterans General Hospital between January 2015 and December 2017. The study conformed with the “Declaration of Helsinki for Medical Research involving Human Subjects.” Additionally, approval was obtained from the institutional review board at Kaohsiung Veterans General Hospital, with the identifier VGHKS18-CT6–09. The study was performed in accordance with approved guidelines.

### Study participants

**Fig. 1:**
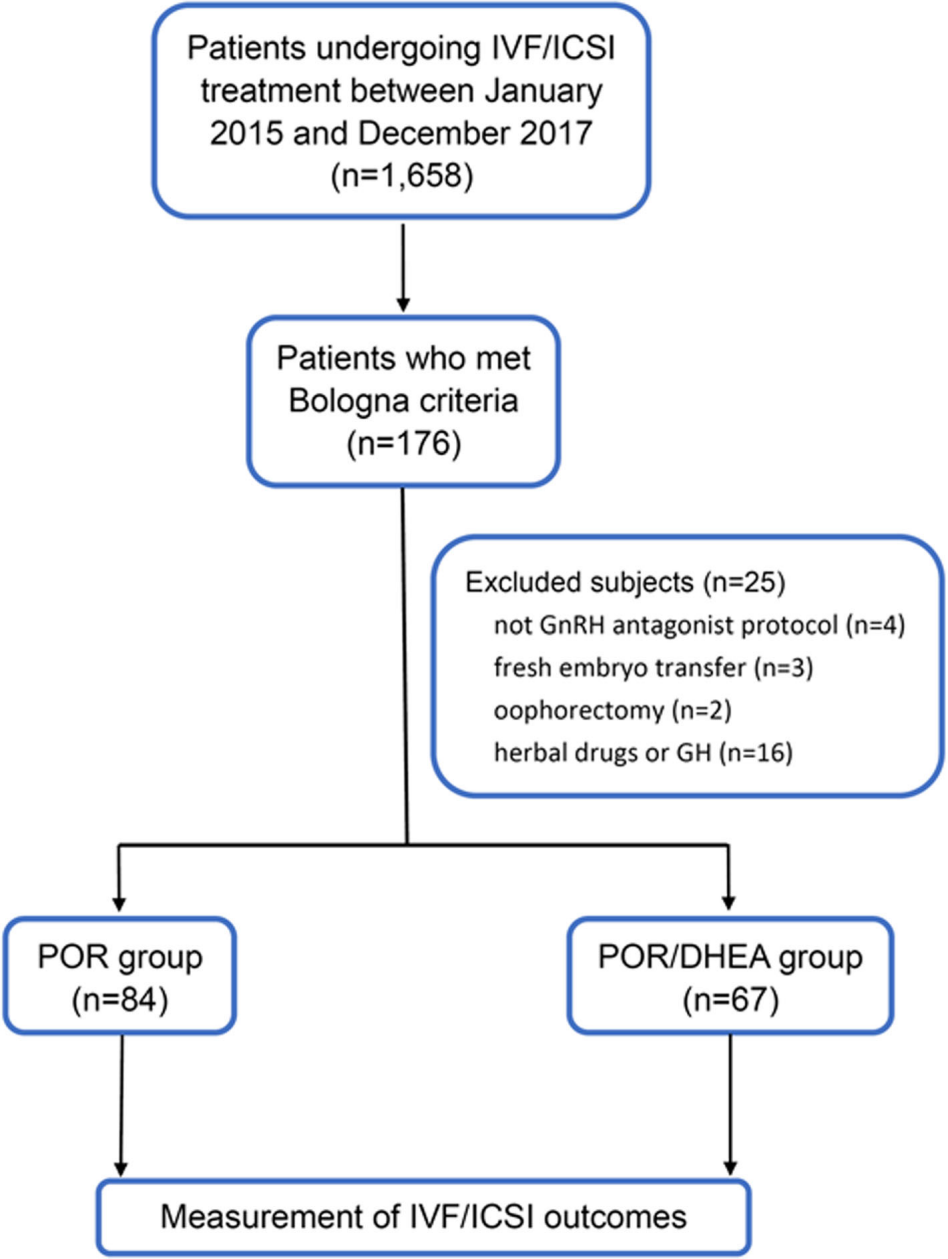
Flow chart of the study design. IVF, in vitro fertilization; ICSI, intracytoplasmic sperm injection; GnRH, gonadotropin-releasing hormone; GH, growth hormone; POR, poor ovarian responder; DHEA, dehydroepiandrosterone

A total of 1658 IVF cycles were performed during the study period. Among these cycles, women who met the Bologna criteria were defined as PORs. Bologna criteria [2] included at least two of the three following features: (1) advanced maternal age (≥ 40 years) or any other risk factor for POR, (2) a previous poor ovarian response (≤ 3 oocytes with a conventional stimulation protocol), and (3) an abnormal ovarian reserve test. An abnormal ovarian reserve test was defined as antral follicle count (AFC) < 5 or anti-Müllerian hormone (AMH) < 1 ng/mL in this study. Additionally, two episodes of a previous POR after maximal stimulation alone would be sufficient to define a patient as a POR. The exclusion criteria were as follows: (1) patients who did not undergo gonadotropin-releasing hormone (GnRH) antagonist protocol, (2) patients who underwent fresh embryo transfer, (3) patients who underwent oophorectomy, and (4) patients who took herbal drugs or other supplementation (e.g., growth hormone). PORs identified in this study were then divided into POR and POR/DHEA groups. In the POR group, patients directly underwent an IVF cycle without DHEA pretreatment. In the POR/DHEA group, patients received daily DHEA supplementation (CPH; Formulation Technology, Oakdale, CA, USA) of 90 mg for an average of 3 months before entry into an IVF cycle. DHEA supplementation or not was decided based on patients’ consideration and preference after full consultation provided by a doctor. The study flow chart is shown in Fig. 1.

### Treatment protocol

The efficacy of IVF protocols, especially between GnRH agonist and antagonist regimens, is still controversial [20, 21]. However, the GnRH antagonist protocol was the most popular one for PORs in a worldwide survey [22]. Therefore, a GnRH antagonist protocol was routinely used for PORs in our reproductive center. Controlled ovarian stimulation was conducted within 5 days of the menstrual cycle, with 300 IU of recombinant FSH (Gonal-F, Merck KGaA, Darmstadt, Germany) or recombinant FSH + LH (Pergovaris, Merck Serono, Aubonne, Switzerland or Merional, Institut Biochimique SA, Lamone, Switzerland). Daily injections of a GnRH antagonist (Cetrotide 0.25 mg, Merck Serono, Idron, France) were given from the day the leading follicle reached 12–14 mm in diameter until the day of oocyte trigger. Dual trigger, combined recombinant hCG (Ovidrel 250 μg, Merck Serono, Modugno, Italy) and GnRH agonist (Lupro 2 mg, Nang Kuang Pharmaceutical Co, Ltd., Tainan, Taiwan), were administered when at least one dominant follicle reached a mean diameter of 17 mm. Oocytes were retrieved 34–36 h after oocyte trigger under the guidance of transvaginal ultrasound. Intracytoplasmic sperm injection (ICSI) was performed in all patients to diminish the potential of fertilization failure. Embryos were evaluated and graded according to the criteria established by the Istanbul consensus workshop [23]. All embryos were cryopreserved by vitrification on the third day after oocyte retrieval for subsequent frozen embryo transfer (FET) cycles. An artificial cycle was used for endometrial preparation of FET, as previously described [24]. Embryo transfer was done under transabdominal sonographic guidance. Regarding luteal phase support, daily progesterone, including Crinone 8% gel (Merck Serono, Hertfordshire, UK) and Duphaston 40 mg (Abbott, Olst, the Netherlands) were given. A pregnancy test was carried out 15 days after embryo transfer. Once a positive pregnancy test was observed, progesterone was continued until 8–10 weeks of gestation.

### Main measurement and outcomes

The primary outcome measures were the number of retrieved oocytes and clinical pregnancy rate. Clinical pregnancy was confirmed if a visible fetal heart beat was found in an intrauterine gestational sac by transvaginal ultrasound. The secondary outcome measures included number of mature oocytes, fertilized oocytes, day 3 embryos, top-quality day 3 embryos, ongoing pregnancy rate and live birth rate. Ongoing pregnancy was defined as the presence of a fetal heart beat beyond 20 weeks of gestation. Live birth was determined by delivery of a live fetus after 20 completed weeks of gestation. Cycle cancellation was defined as incomplete cycles due to no response to gonadotropins or no retrieved oocyte.

### Statistical analysis

Normality of quantitative variables was revealed by Kolmogorov-Smirnov test. Independent t-tests were used to compare quantitative variables. The categorical variables were compared using Chi-Square tests. In addition, binary logistic regression was used to assess odds ratios (ORs) and 95% confidence intervals (CIs) of clinical pregnancy and the number of retrieved oocytes > 3 after adjusting for confounders. All analyses were performed using the Statistical Package for Social Sciences (SPSS) version 20.0 (Chicago, IL, USA). Comparisons with a *p*-value less than 0.05 were considered significant.

## Results

A total of 176 cycles met the Bologna criteria (10.6%). Among these cycles, there were 4 cycles that did not receive GnRH antagonist protocol, 3 cycles that received fresh embryo transfer, 2 cycles that underwent oophorectomy, and 16 cycles that received herbal drugs or growth hormone and were excluded from the study. Ultimately, 151 cycles were identified in this study and divided into the POR group (*n* = 84) and the POR/DHEA group (*n* = 67). There were no differences between the two groups regarding age, body mass index, infertility duration, previous IVF attempts, primary or secondary infertility, basal follicle stimulation hormone (FSH), dehydroepiandrosterone-sulfate (DHEA-S) concentration, AFC, AMH and Bologna criteria category (Table 1).

**Table 1:** Basic characteristics of poor ovarian responders with or without DHEA

Parameters	POR (*n* = 84)	POR with DHEA (*n* = 67)	*p* value
Age (years)	39.8 ± 3.7	39.1 ± 3.3	0.208
Body mass index (kg/m2)	21.8 ± 3.8	21.9 ± 2.9	0.857
Infertility duration (year)	5.3 ± 4.5	5.6 ± 4.2	0.712
Previous IVF attempts(n)	2.3 ± 2.2	2.6 ± 2.2	0.480
Types of infertility n (%)			0.548
Primary infertility	41/84 (48.8%)	36/67 (53.7%)	
Secondary infertility	43/84 (51.2%)	31/67 (46.3%)	
Basal FSH (IU/l)	6.3 ± 4.6	7.1 ± 4.2	0.305
DHEA-S (μg/dl)	212.9 ± 85.9	199.2 ± 65.0	0.316
Antral follicle counts (n)	3.3 ± 1.4	3.3 ± 1.3	0.921
Anti-Müllerian hormone(ng/ml)	0.7 ± 0.4	0.7 ± 0.5	0.932
^a^Bologna criteria category n (%)			0.170
1 + 2	16/84 (19.0%)	5/67 (7.5%)	
1 + 3	16/84 (19.0%)	19/67 (28.4%)	
2 + 3	21/84 (25.0%)	17/67 (25.4%)	
1 + 2 + 3	31/84 (36.9%)	26/67 (38.8%)	

Data are presented as mean ± standard deviation and n (%)

*POR* poor ovarian responder, *DHEA* dehydroepiandrosterone, *IVF* in vitro fertilization, *FSH* follicle stimulation hormone, *DHEA-S* dehydroepiandrosterone-sulfate

^a^PORs meet the Bologna criteria, having at least two of the three following features: (1) advanced maternal age (≥ 40 years) or any other risk factor for POR; (2) a previous POR (≤ 3 oocytes with a conventional stimulation protocol); (3) an abnormal ovarian reserve test. Abnormal ovarian reserve test was defined as antral follicle count (AFC) < 5 or anti-Müllerian hormone (AMH) < 1 ng/mL in this study

The cycle characteristics and IVF outcomes between the two groups are presented in Table 2. There were no significant differences in terms of stimulation duration or gonadotropin dose. The POR/DHEA group had a greater number of retrieved oocytes (3.3 ± 2.5 vs. 2.0 ± 1.5, *p* < 0.001), metaphase II oocytes (1.9 ± 1.5 vs. 1.0 ± 1.1, *p* < 0.001), fertilized oocytes (2.3 ± 1.7 vs. 1.4 ± 1.2, *p* < 0.001), day 3 embryos (2.1 ± 1.6 vs. 1.3 ± 1.2, *p* = 0.001) and top-quality embryos at day 3 (1.0 ± 1.1 vs. 0.4 ± 0.8, *p* = 0.001) than did the POR group. Furthermore, the rate of retrieved oocytes > 3 (34.3% vs. 14.3%, *p* = 0.004), the clinical pregnancy rate (23.9% vs. 7.1%, *p* = 0.004), ongoing pregnancy rate (17.9% vs. 6.0%, *p* = 0.021) and live birth rate (16.4% vs. 6.0%, *p* = 0.038) were significantly higher in the POR/DHEA group than in the POR group. Cycle cancellation rate was similar between the two groups.

**Table 2:** Cycle characteristics and pregnancy outcome of poor ovarian responders with or without DHEA

Parameters	POR (*n* = 84)	POR with DHEA (*n* = 67)	*p* value
Stimulation duration (days)	10.1 ± 2.2	10.5 ± 1.9	0.310
Gonadotropin dosage (IU)	2884.4 ± 872.5	2975.4 ± 649.4	0.126
No. of oocytes retrieved (n)	2.0 ± 1.5	3.3 ± 2.5	< 0.001
Oocytes retrieved > 3, % (n)	14.3 (12/84)	34.3 (23/67)	0.004
No. of metaphase II oocytes (n)	1.0 ± 1.1	1.9 ± 1.5	< 0.001
Maturation rate (%)	35.5 ± 36.2	56.2 ± 33.9	< 0.001
No. of fertilized oocytes (n)	1.4 ± 1.2	2.3 ± 1.7	< 0.001
Fertilization rate (%)	58.0 ± 42.5	67.9 ± 32.7	0.108
No. of Day 3 embryos (n)	1.3 ± 1.2	2.1 ± 1.6	0.001
No. of top-quality Day 3 embryos (n)	0.4 ± 0.8	1.0 ± 1.1	0.001
Clinical pregnancy rate, % (n)	7.1 (6/84)	23.9 (16/67)	0.004
Ongoing pregnancy rate, % (n)	6.0 (5/84)	17.9 (12/67)	0.021
Live birth rate, % (n)	6.0 (5/84)	16.4 (11/67)	0.038
Cancellation rate, % (n)	16.7 (14/84)	10.4 (7/67)	0.273

Data are presented as mean ± standard deviation and % (n)

*POR* poor ovarian responder, *DHEA* dehydroepiandrosterone

As shown in Table 3, the multivariate analysis revealed that DHEA supplementation was significantly associated with clinical pregnancy rate (OR = 4.93, 95% CI 1.68–14.53, *p* = 0.004). In addition, in the POR/DHEA group, multivariate analysis (Table 4) showed POR with serum concentration of DHEA-S less than 180 μg/dl was significantly associated with the possibility to retrieve more than 3 oocytes (OR = 5.92, 95% CI 1.48–23.26, *p* = 0.012).

**Table 3:** Analyses of factors affecting clinical pregnancy rate in poor ovarian responders

	Univariate analysis		Multivariate analysis	
	OR (95% CI)	*p* value	Adjusted OR (95% CI)	*p* value
DHEA				
Yes vs. No	4.08(1.50–11.11)	0.006	4.93(1.68–14.53)	0.004
Age (years)	0.91(0.81–1.02)	0.114	0.95(0.84–1.09)	0.466
BMI (kg/m2)	1.05(0.93–1.20)	0.397		
Infertility duration	0.90(0.79–1.03)	0.120	0.88(0.76–1.03)	0.106
Previous IVF attempts (n)	0.95(0.77–1.20)	0.670		
Types of infertility				
Primary vs. Secondary	1.05(0.42–2.59)	0.920		
Basal FSH (IU/l)	1.04(0.94–1.15)	0.435		
AFC (n)	1.56(1.11–2.21)	0.011	1.62(1.11–2.37)	0.012
AMH (ng/ml)	1.44(0.52–4.00)	0.484		
^a^Bologna criteria category				
(1 + 3) vs. (1 + 2)	4.14(0.46–37.06)	0.204		
(2 + 3) vs. (1 + 2)	5.33(0.62–46.00)	0.128		
(1 + 2 + 3) vs. (1 + 2)	2.80(0.32–24.24)	0.350		

*OR* odd ratio, *CI* confidence interval, *DHEA* dehydroepiandrosterone, *BMI* body mass index, *IVF* in vitro fertilization, *FSH* follicle stimulation hormone, *AFC* antral follicle counts, *AMH* anti-Müllerian hormone

^a^Poor ovarian responders (PORs) meet the Bologna criteria, having at least two of the three following features: (1) advanced maternal age (≥ 40 years) or any other risk factor for POR; (2) a previous POR (≤ 3 oocytes with a conventional stimulation protocol); (3) an abnormal ovarian reserve test. Abnormal ovarian reserve test was defined as antral follicle count (AFC) < 5 or anti-Müllerian hormone (AMH) < 1 ng/mL in this study

**Table 4:** Analyses of factors affecting retrieved oocytes > 3 in poor ovarian responders with DHEA supplementation

	Univariate analysis		Multivariate analysis	
	OR (95% CI)	*p* value	Adjusted OR (95% CI)	*p* value
DHEA-S, μg/dl				
< 180 vs. ≥ 180	6.62(1.90–22.73)	0.003	5.92(1.48–23.26)	0.012
Age, years	0.94(0.80–1.09)	0.404		
BMI, kg/m2	1.00(0.84–1.20)	0.986		
Infertility duration	1.03(0.92–1.16)	0.620		
Previous IVF attempts, n	1.07(0.86–1.34)	0.541		
Types of infertility				
Primary vs. Secondary	1.81(0.65–5.02)	0.258		
^a^Bologna criteria category				
(1 + 3) vs. (1 + 2)	0.09(0.01–1.00)	0.050	0.22(0.02–2.92)	0.253
(2 + 3) vs. (1 + 2)	0.18(0.02–1.92)	0.154	0.43(0.03–6.05)	0.530
(1 + 2 + 3) vs. (1 + 2)	0.09(0.01–0.97)	0.047	0.17(0.01–2.05)	0.164

*DHEA* dehydroepiandrosterone, *OR* odd ratio, *CI* confidence interval, *DHEA-S* dehydroepiandrosterone-sulfate*, BMI* body mass index, *IVF* in vitro fertilization

^a^Poor ovarian responders (PORs) meet the Bologna criteria, having at least two of the three following features: (1) advanced maternal age (≥ 40 years) or any other risk factor for POR; (2) a previous POR (≤ 3 oocytes with a conventional stimulation protocol); (3) an abnormal ovarian reserve test. Abnormal ovarian reserve test was defined as antral follicle count (AFC) < 5 or anti-Müllerian hormone (AMH) < 1 ng/mL in this study

## Discussion

In this retrospective cohort study, the incidence rate of POR based on Bologna criteria was 10.6% (176/1658) and was compatible with that of previous study [25]. Additionally, the poor prognosis of Bologna PORs undergoing IVF/ICSI cycles without DHEA supplementation in this study was also consistent with previous study [3]. However, after DHEA pretreatment, the present study demonstrated that number of retrieved oocytes, metaphase II oocytes, fertilized oocytes, day 3 embryos, top-quality embryos at day 3 as well as clinical pregnancy rate, ongoing pregnancy rate and live birth rate significantly improved. Furthermore, the multivariate analysis displayed that PORs with DHEA supplementation exhibited a 4.93-fold increase in the clinical pregnancy rate (95% CI 1.68–14.53, *p* = 0.004) compared to those without DHEA supplementation (Table 3). Numerous studies [15, 17, 26–28] and some meta-analyses [18, 19, 29] also support the beneficial effects of DHEA on PORs undergoing IVF/ICSI cycles. The systematic review and meta-analysis conducted by Zhang et al. showed that DHEA supplementation increased the clinical pregnancy rate, live birth rate and ovarian reserve in patients with poor ovarian response [18]. However, few randomized controlled trials with limited cases were analyzed, and the inclusive criteria for PORs were heterogeneous. Moreover, the Cochrane Review included only randomized controlled trials and concluded that pretreatment with DHEA may be associated with improved live birth rate in patients undergoing IVF/ICSI cycles [19]. Nevertheless, the Cochrane Review also included the studies of pretreatment with testosterone and the patients who were not identified as PORs. An updated randomized controlled trial performed by Kotb and colleagues, enrolled PORs according to the Bologna criteria and revealed that DHEA supplementation significantly increased the number of oocytes, fertilization rate, fertilized oocytes, clinical pregnancy rate and ongoing pregnancy rate [26]. Similarly, the small sample size was also a limitation of the study. Therefore, taken together, based on current evidence and our retrospective cohort study, DHEA supplementation seems to improve IVF/ICSI outcomes and ovarian reserve in PORs. However, additional large-scale, well-designed randomized controlled trials are necessary to confirm the beneficial role of DHEA in PORs. Furthermore, although we used a GnRH antagonist protocol in this study, we believed that other protocols, especially a non-suppressive stimulation protocol, like microcode agonist protocol could also reach better results after DHEA supplementation. However, further studies are needed to prove this concept.

The major mechanism of DHEA on the improvement of reproductive outcomes is associated with increased androgen after DHEA supplementation. DHEA, a precursor of estradiol and testosterone, serves as a prohormone of follicular fluid testosterone during ovarian induction [30]. Androgen receptors (ARs) have been identified in the granulosa cells (GCs) at any follicular stage, especially preantral and antral follicles [31, 32]. In GC-specific AR knockout mice, mice were subfertile with longer estrous cycles, fewer ovulated oocytes, reduced follicle progression and increased follicle atresia [33, 34]. GC-specific ARs seemed to be pivotal regulators of follicular development and fertility. Indeed, androgen has been reported to play roles in recruitment and initiation of primordial follicles [35, 36], promotion of follicular growth by increasing FSH receptor expression [37, 38], and prevention of follicular atresia by reducing apoptosis [37]. Moreover, DHEA administration increases serum concentration of insulin-like growth factor-1 (IGF-1) [39], which has been reported to be correlated with oocyte quality and embryo development [40, 41]. Therefore, indirect action of DHEA was mainly presented. However, direct action of DHEA on the target organs has been proposed [42, 43] but is still inconclusive. Regarding the molecular mechanism, our previous studies revealed that DHEA supplementation could improve mitochondrial function and reduce apoptosis in the cumulus cells [44] and human granulosa cell line [45].

Returning to clinical practice, however, not all PORs benefit from the DHEA pretreatment. Thus, we attempted to investigate which subgroups of PORs with DHEA supplementation benefit most from such pretreatment. The current study found that PORs with lower DHEA-S concentration (< 180 μg/dl) exhibited a 5.92-fold increase in the rate of retrieved oocytes > 3 (95% CI 1.48–23.26, *p* = 0.012) compared to those with higher DHEA-S concentration (≥ 180 μg/dl) (Table 4). Hypoandrogenism has been reported to be in association with diminished ovarian reserve [46]. Low androgen levels can be of ovarian and/or adrenal etiology. Since DHEA-S is almost exclusively produced by adrenals, it is generally accepted that low DHEA-S reflects an adrenal cause for low androgen levels. Therefore, our finding would suggest that DHEA supplementation was especially effective if androgen deficiency was of adrenal origin. Gleicher et al. demonstrated that patients with secondary ovarian insufficiency induced by adrenal hypoandrogenism dramatically improved in ovarian function after DHEA supplementation [47]. Another study conducted by Gleicher and colleagues showed that in women with high-AMH/low-testosterone phenotype associated with adrenal insufficiency, DHEA supplementation equalizes low to normal testosterone and normalizes IVF cycle outcomes [48]. Taken together, patients with low DHEA-S levels, implying adrenal hypoandrogenism, could obtain greater improvement from DHEA supplementation. In addition, in developed countries, most cases of primary adrenal insufficiency are caused by autoimmunity, frequently coexisting with other autoimmune abnormalities [49]. Therefore, further survey for autoimmunity in these patients may be needed.

The strength of this study lies in its strict exclusion criteria and subgroup analysis. However, there were some limitations in this study. First, this is a retrospective study with relatively small sample size. Second, the patients identified based on the Bologna criteria may be heterogeneous. Moreover, serum DHEA-S levels were checked not at the time when patients were included in the study, but within 6 months before DHEA supplementation or IVF cycles. We believed that the serum DHEA-S levels of this study were representative. However, potential bias could not be excluded.

## Conclusion

In conclusion, the IVF outcomes of PORs could potentially benefit from pretreatment with DHEA. In PORs who received DHEA supplementation, women with lower DHEA-S levels could achieve higher possibility of retrieving more than 3 oocytes.

## Abbreviations

AFC: Antral follicle count
AMH: Anti-Müllerian hormone
AR: Androgen receptor
BMI: Body mass index
CI: Confidence interval
DHEA: Dehydroepiandrosterone
DHEA*-*S: Dehydroepiandrosterone-sulfate
FET: Frozen embryo transfer
FSH: Follicle stimulation hormone
GC: Granulosa cell
GnRH: Gonadotropin-releasing hormone
ICSI: Intracytoplasmic sperm injection
IGF-1: Insulin-like growth factor-1
IVF: In vitro fertilization
OR: Odd ratio
POR: Poor ovarian responder

## References

[1] Polyzos NP, Devroey P (2011). A systematic review of randomized trials for the treatment of poor ovarian responders: is there any light at the end of the tunnel?. Fertil Steril.

[2] Ferraretti AP, La Marca A, Fauser BC, Tarlatzis B, Nargund G, Gianaroli L (2011). ESHRE consensus on the definition of ‘poor response’ to ovarian stimulation for in vitro fertilization: the Bologna criteria. Hum Reprod.

[3] Polyzos NP, Nwoye M, Corona R, Blockeel C, Stoop D, Haentjens P (2014). Live birth rates in Bologna poor responders treated with ovarian stimulation for IVF/ICSI. Reprod BioMed Online.

[4] Xu B, Chen Y, Geerts D, Yue J, Li Z, Zhu G (2018). Cumulative live birth rates in more than 3,000 patients with poor ovarian response: a 15-year survey of final in vitro fertilization outcome. Fertil Steril.

[5] Pandian Z, McTavish AR, Aucott L, Hamilton MP, Bhattacharya S. Interventions for ‘poor responders’ to controlled ovarian hyper stimulation (COH) in in-vitro fertilisation (IVF). Cochrane Database Syst Rev. 2010;(1):Cd004379. 10.1002/14651858.CD004379.pub3 PMC13243861 20091563

[6] Sunkara SK, Coomarasamy A, Faris R, Braude P, Khalaf Y (2014). Long gonadotropin-releasing hormone agonist versus short agonist versus antagonist regimens in poor responders undergoing in vitro fertilization: a randomized controlled trial. Fertil Steril.

[7] Bosdou JK, Venetis CA, Kolibianakis EM, Toulis KA, Goulis DG, Zepiridis L (2012). The use of androgens or androgen-modulating agents in poor responders undergoing in vitro fertilization: a systematic review and meta-analysis. Hum Reprod Update.

[8] Li XL, Wang L, Lv F, Huang XM, Wang LP, Pan Y (2017). The influence of different growth hormone addition protocols to poor ovarian responders on clinical outcomes in controlled ovary stimulation cycles: a systematic review and meta-analysis. Medicine (Baltimore).

[9] Chamayou S, Sicali M, Alecci C, Ragolia C, Liprino A, Nibali D (2017). The accumulation of vitrified oocytes is a strategy to increase the number of euploid available blastocysts for transfer after preimplantation genetic testing. J Assist Reprod Genet.

[10] Burger HG (2002). Androgen production in women. Fertil Steril.

[11] Prizant H, Gleicher N, Sen A (2014). Androgen actions in the ovary: balance is key. J Endocrinol.

[12] Casson PR, Lindsay MS, Pisarska MD, Carson SA, Buster JE (2000). Dehydroepiandrosterone supplementation augments ovarian stimulation in poor responders: a case series. Hum Reprod.

[13] Singh N, Zangmo R, Kumar S, Roy KK, Sharma JB, Malhotra N (2013). A prospective study on role of dehydroepiandrosterone (DHEA) on improving the ovarian reserve markers in infertile patients with poor ovarian reserve. Gynecol Endocrinol.

[14] Barad D, Gleicher N (2006). Effect of dehydroepiandrosterone on oocyte and embryo yields, embryo grade and cell number in IVF. Hum Reprod.

[15] Zangmo R, Singh N, Kumar S, Vanamail P, Tiwari A (2014). Role of dehydroepiandrosterone in improving oocyte and embryo quality in IVF cycles. Reprod BioMed Online.

[16] Barad D, Brill H, Gleicher N (2007). Update on the use of dehydroepiandrosterone supplementation among women with diminished ovarian function. J Assist Reprod Genet.

[17] Wiser A, Gonen O, Ghetler Y, Shavit T, Berkovitz A, Shulman A (2010). Addition of dehydroepiandrosterone (DHEA) for poor-responder patients before and during IVF treatment improves the pregnancy rate: a randomized prospective study. Hum Reprod.

[18] Zhang M, Niu W, Wang Y, Xu J, Bao X, Wang L (2016). Dehydroepiandrosterone treatment in women with poor ovarian response undergoing IVF or ICSI: a systematic review and meta-analysis. J Assist Reprod Genet.

[19] Nagels HE, Rishworth JR, Siristatidis CS, Kroon B (2015). Androgens (dehydroepiandrosterone or testosterone) for women undergoing assisted reproduction. Cochrane Database Syst Rev.

[20] Orvieto R, Patrizio P (2013). GnRH agonist versus GnRH antagonist in ovarian stimulation: an ongoing debate. Reprod BioMed Online.

[21] Lambalk CB, Banga FR, Huirne JA, Toftager M, Pinborg A, Homburg R (2017). GnRH antagonist versus long agonist protocols in IVF: a systematic review and meta-analysis accounting for patient type. Hum Reprod Update.

[22] Patrizio P, Vaiarelli A, Levi Setti PE, Tobler KJ, Shoham G, Leong M (2015). How to define, diagnose and treat poor responders? Responses from a worldwide survey of IVF clinics. Reprod BioMed Online.

[23] Alpha Scientists in Reproductive Medicine and ESHRE Special Interest Group of Embryology (2011). The Istanbul consensus workshop on embryo assessment: proceedings of an expert meeting. Hum Reprod.

[24] Zheng Y, Dong X, Huang B, Zhang H, Ai J (2015). The artificial cycle method improves the pregnancy outcome in frozen-thawed embryo transfer: a retrospective cohort study. Gynecol Endocrinol.

[25] Yang S, Chen X, Zhen X, Wang H, Ma C, Li R (2015). The prognosis of IVF in poor responders depending on the Bologna criteria: a large sample retrospective study from China. Biomed Res Int.

[26] Kotb MM, Hassan AM, AwadAllah AM (2016). Does dehydroepiandrosterone improve pregnancy rate in women undergoing IVF/ICSI with expected poor ovarian response according to the Bologna criteria? A randomized controlled trial. Eur J Obstet Gynecol Reprod Biol.

[27] Xu B, Li Z, Yue J, Jin L, Li Y, Ai J (2014). Effect of dehydroepiandrosterone administration in patients with poor ovarian response according to the Bologna criteria. PLoS One.

[28] Jirge PR, Chougule SM, Gavali VG, Bhomkar DA (2014). Impact of dehydroepiandrosterone on clinical outcome in poor responders: a pilot study in women undergoing in vitro fertilization, using bologna criteria. J Hum Reprod Sci.

[29] Li J, Yuan H, Chen Y, Wu H, Wu H, Li L (2015). A meta-analysis of dehydroepiandrosterone supplementation among women with diminished ovarian reserve undergoing in vitro fertilization or intracytoplasmic sperm injection. Int J Gynaecol Obstet.

[30] Haning RV,J, Hackett RJ, Flood CA, Loughlin JS, Zhao QY, Longcope C (1993). Plasma dehydroepiandrosterone sulfate serves as a prehormone for 48% of follicular fluid testosterone during treatment with menotropins. J Clin Endocrinol Metab.

[31] Hillier SG, Tetsuka M, Fraser HM (1997). Location and developmental regulation of androgen receptor in primate ovary. Hum Reprod.

[32] Slomczynska M, Tabarowski Z (2001). Localization of androgen receptor and cytochrome P450 aromatase in the follicle and corpus luteum of the porcine ovary. Anim Reprod Sci.

[33] Sen A, Hammes SR (2010). Granulosa cell-specific androgen receptors are critical regulators of ovarian development and function. Mol Endocrinol.

[34] Walters KA, Middleton LJ, Joseph SR, Hazra R, Jimenez M, Simanainen U (2012). Targeted loss of androgen receptor signaling in murine granulosa cells of preantral and antral follicles causes female subfertility. Biol Reprod.

[35] Smith P, Steckler TL, Veiga-Lopez A, Padmanabhan V (2009). Developmental programming: differential effects of prenatal testosterone and dihydrotestosterone on follicular recruitment, depletion of follicular reserve, and ovarian morphology in sheep. Biol Reprod.

[36] Magamage MPS, Zengyo M, Moniruzzaman M, Miyano T (2011). Testosterone induces activation of porcine primordial follicles in vitro. Reprod Med Biol.

[37] Sen A, Prizant H, Light A, Biswas A, Hayes E, Lee HJ (2014). Androgens regulate ovarian follicular development by increasing follicle stimulating hormone receptor and microRNA-125b expression. Proc Natl Acad Sci U S A.

[38] Laird M, Thomson K, Fenwick M, Mora J, Franks S, Hardy K (2017). Androgen stimulates growth of mouse Preantral follicles in vitro: interaction with follicle-stimulating hormone and with growth factors of the TGFbeta superfamily. Endocrinology.

[39] Genazzani AD, Stomati M, Strucchi C, Puccetti S, Luisi S, Genazzani AR (2001). Oral dehydroepiandrosterone supplementation modulates spontaneous and growth hormone-releasing hormone-induced growth hormone and insulin-like growth factor-1 secretion in early and late postmenopausal women. Fertil Steril.

[40] Fried G, Remaeus K, Harlin J, Krog E, Csemiczky G, Aanesen A (2003). Inhibin B predicts oocyte number and the ratio IGF-I/IGFBP-1 may indicate oocyte quality during ovarian hyperstimulation for in vitro fertilization. J Assist Reprod Genet.

[41] Liu HC, He ZY, Mele CA, Veeck LL, Davis O, Rosenwaks Z (1999). Human endometrial stromal cells improve embryo quality by enhancing the expression of insulin-like growth factors and their receptors in cocultured human preimplantation embryos. Fertil Steril.

[42] Alexaki VI, Charalampopoulos I, Panayotopoulou M, Kampa M, Gravanis A, Castanas E (2009). Dehydroepiandrosterone protects human keratinocytes against apoptosis through membrane binding sites. Exp Cell Res.

[43] Liu D, Si H, Reynolds KA, Zhen W, Jia Z, Dillon JS (2007). Dehydroepiandrosterone protects vascular endothelial cells against apoptosis through a Galphai protein-dependent activation of phosphatidylinositol 3-kinase/Akt and regulation of antiapoptotic Bcl-2 expression. Endocrinology.

[44] Lin LT, Wang PH, Wen ZH, Li CJ, Chen SN, Tsai EM (2017). The application of Dehydroepiandrosterone on improving mitochondrial function and reducing apoptosis of cumulus cells in poor ovarian responders. Int J Med Sci.

[45] Tsui KH, Wang PH, Lin LT, Li CJ (2017). DHEA protects mitochondria against dual modes of apoptosis and necroptosis in human granulosa HO23 cells. Reproduction.

[46] Gleicher N, Kim A, Weghofer A, Kushnir VA, Shohat-Tal A, Lazzaroni E (2013). Hypoandrogenism in association with diminished functional ovarian reserve. Hum Reprod.

[47] Gleicher N, Kushnir VA, Weghofer A, Barad DH (2016). The importance of adrenal hypoandrogenism in infertile women with low functional ovarian reserve: a case study of associated adrenal insufficiency. Reprod Biol Endocrinol.

[48] Gleicher N, Kushnir VA, Darmon SK, Wang Q, Zhang L, Albertini DF (2017). New PCOS-like phenotype in older infertile women of likely autoimmune adrenal etiology with high AMH but low androgens. J Steroid Biochem Mol Biol.

[49] Charmandari E, Nicolaides NC, Chrousos GP (2014). Adrenal insufficiency. Lancet.

